# Supplementary material for: Inferring residue level hydrogen deuterium exchange with ReX
Source: Commun Chem. 2025 Nov 10;8:343. doi: 10.1038/s42004-025-01719-4 (PMC12603136; doi:10.1038/s42004-025-01719-4)
Supplement: Supplementary file 2 — Supplementary Manuscript [file 42004_2025_1719_MOESM2_ESM.pdf]

Supplementary information to: Inferring residue level hydrogen deuterium exchange with ReX

Oliver M. Crook * [1,2], Nathan Gittens[3], Chun-wa Chung[3], and Charlotte M. Deane[4]

[1] *Department of Chemistry, Dorothy Crowfoot Hodgkin Building, University of Oxford, Oxford, UK*
[2] *Kavli Institute for Nanoscience Discovery, University of Oxford, Oxford, UK*
[3] *Department of Statistics, University of Oxford, Oxford, UK*
[4] *Structural and Biophysical Sciences, GlaxoSmithKline R&D, Stevenage, UK*

September 1, 2025

*oliver.crook@stats.ox.ac.uk

1 Additional Benchmarking Results

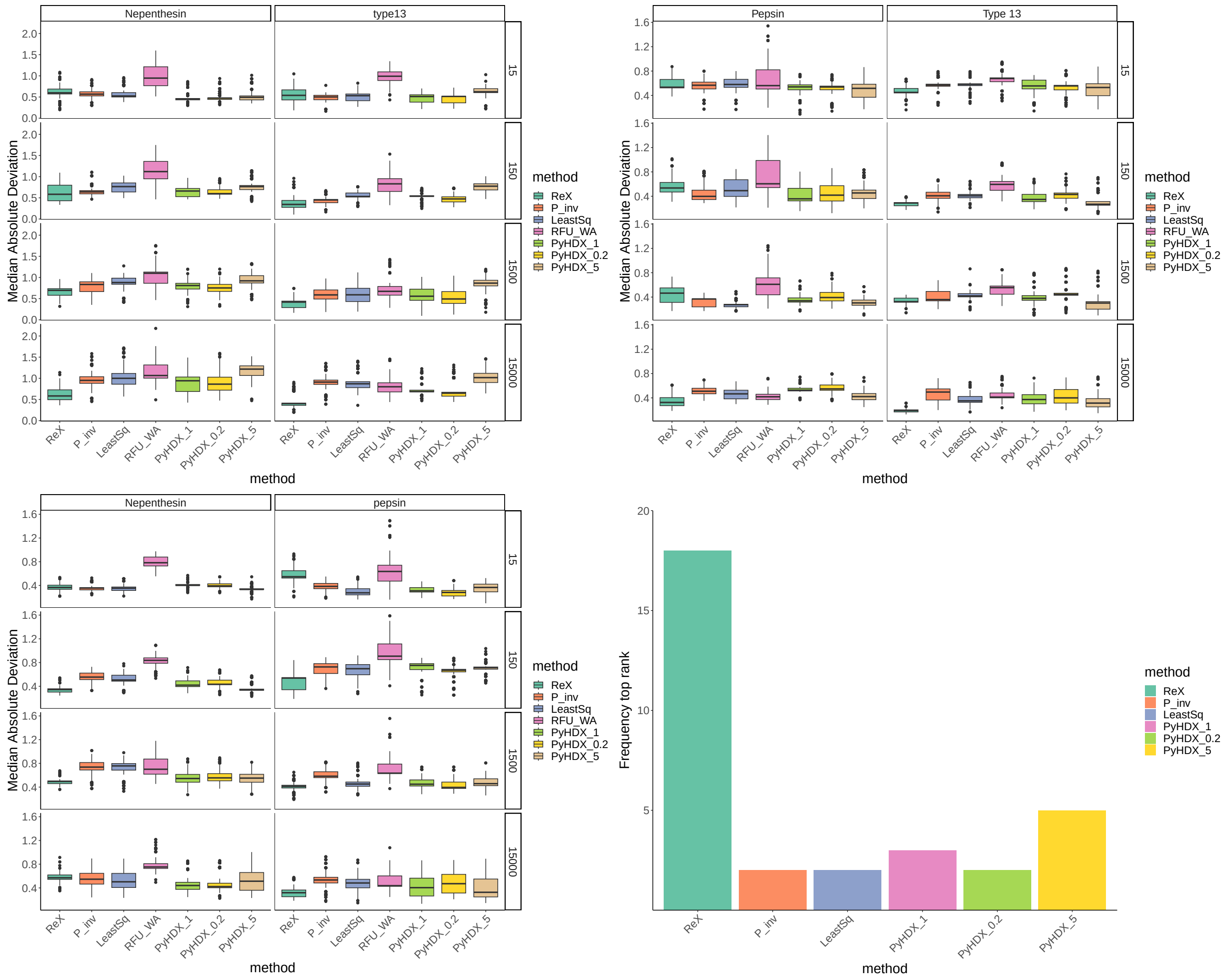

Figure S1: **Additional Benchmarking results.** Benchmarking results comparing different residue resolved approaches. Boxpots are in the style of Tukey presenting approximate 95% confidence intervals. The boxplots represent bootstrap distributions of the Median Absolute Deviation (MAD) of the predicted and observed values. (Upper Left) trained on Pepsin (IUpper Right) Trained on Nepenthesin (downsampled to 40 peptides) (Lower Left) trained on aspergillus pepsin type XIII. (Bottom Right) Summary of results.

2 Comparison of Rex with NMR Measurements

To our knowledge, only one dataset exists that contains both residue-level NMR measurements and HDX-MS measurements with fully deuterated controls, on the mouse prion protein (Moulick *et al.*, 2015; Stofella *et al.*, 2022). This dataset is limited in scope, containing only 14 MS peptides and 27 NMR residues across a 101-residue protein, with no replicates and low redundancy (average = 1, maximum = 3).

We applied ReX to the MS dataset and compared the inferred residue-level uptakes to the experimental NMR measurements. We observed several contradictions between the two modalities. In particular, for $\sim 10\%$ of residues the summed NMR uptakes exceeded the corresponding MS peptide uptakes, implying that the remaining unmeasured residues would require negative uptake to be compatible. Stofella *et al.* (2022) excluded three outlier residues to report a correlation of 0.71. ReX achieved a correlation of 0.72 without excluding outliers, although coverage analysis indicated that a small number of residues were poorly modelled.

Calibration of ReX's Bayesian credible bands showed average coverage of 91%, slightly anti-conservative but reasonable given the contradictions (Fig. S2). Notably, the residues with poor coverage did not align with those identified as outliers by Stofella *et al.* (2022). Instead, discrepancies were concentrated in regions with low redundancy or contradictory peptide measurements (Fig. S3).

These results suggest that benchmarking residue-level methods on this dataset is problematic: models that adhere closely to the MS data inevitably contradict the NMR data, and vice versa. We therefore did not include this dataset in our benchmarking framework. Nonetheless, this analysis demonstrates that ReX performs robustly under conditions of conflicting input data, and could in future be extended to incorporate NMR-derived constraints directly.

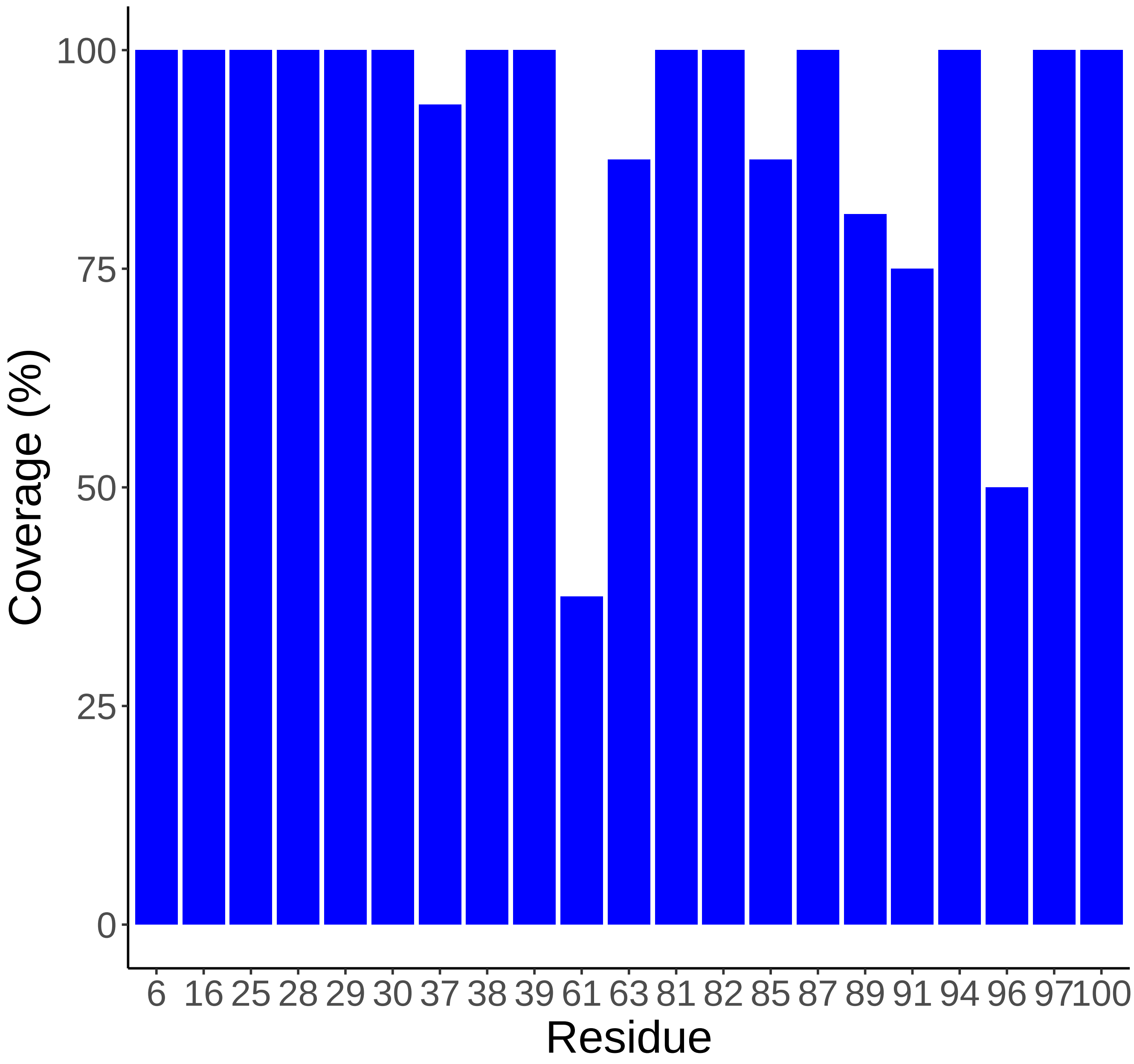

Figure S2: **Rex coverage on mouse prion data** Percentage of NMR measurements falling within ReX's residue-level 95% credible intervals.

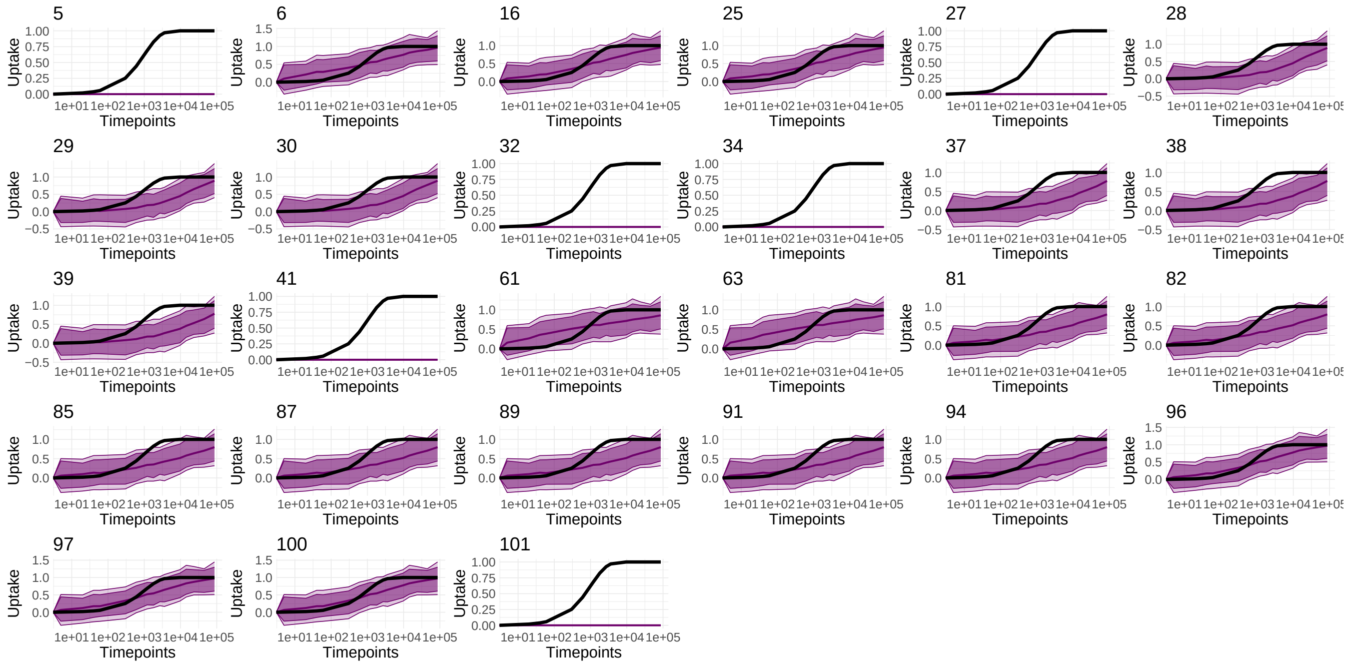

Figure S3: **ReX uptake inference compared with NMR data.** NMR uptake curves (black) overlaid with ReX residue-level credible intervals inferred from MS data..

3 Error Distributions

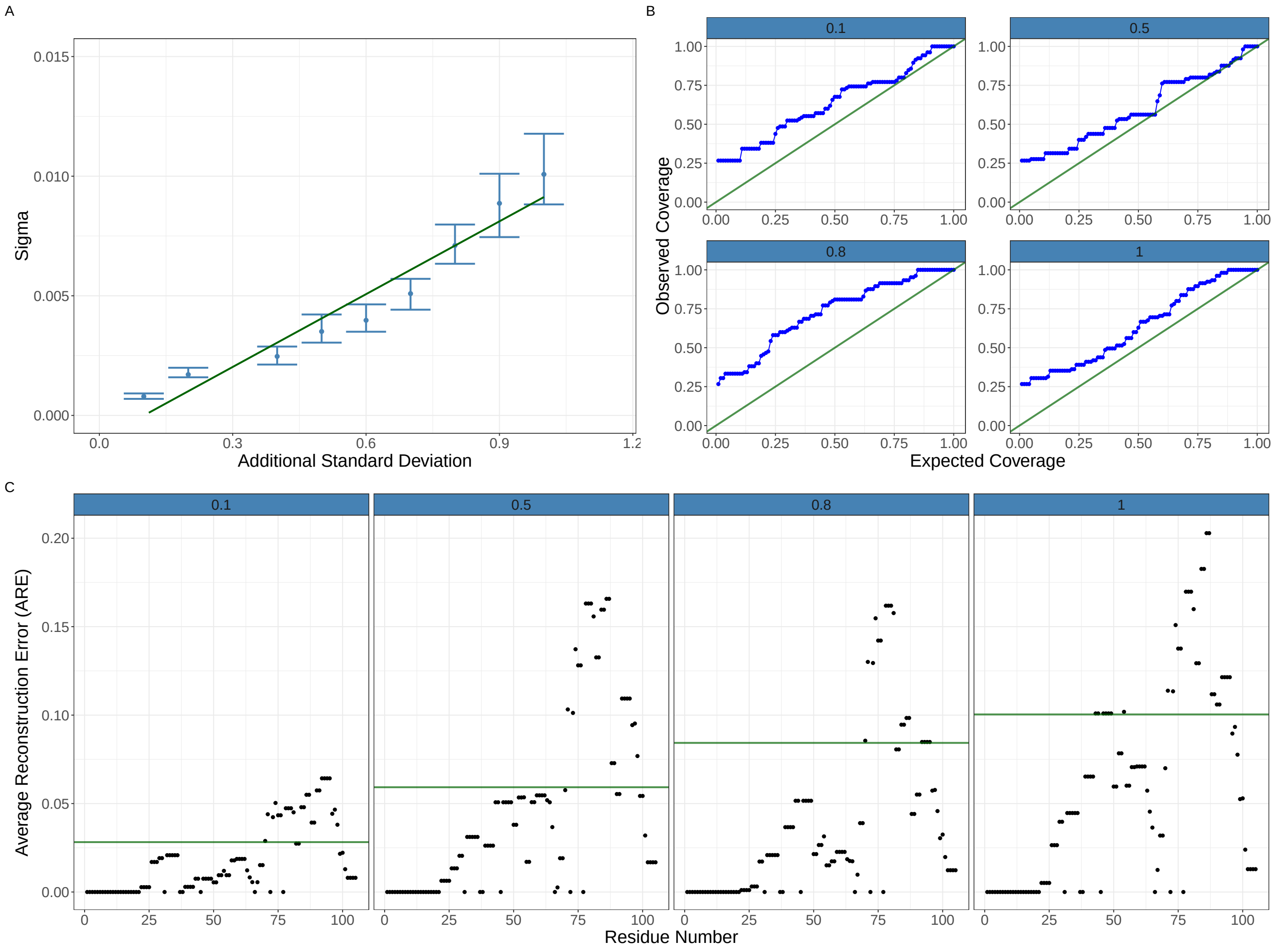

Figure S4: **Error distribution Figure.** Plots representing how adding additional standard deviations effect the quality measures. (A) The added additional standard deviation and the inferred Sigma value. Equitailed 95% credible interval reported. Straight line fitted to data demonstrated close to linear increasing relationship between the additional standard deviation and the inferred Sigma. (B) Coverage plots showing that credible interval constructed using Sigma overestimate the variability in the data and are hence conservative. Plots are faceted by additional standard deviation. (C) Average Reconstruction Error (ARE) plotted against Residue Number. The dark green line is the inferred median value of the square root of Sigma and the plots are faceted by additional standard deviation. ARE and Sigma are faithful to each other and the additional standard deviation.

4 Reference parameters for minimum detectable effects

The inferred value of Sigma allows us to deduce minimal detectable effects in deuterium difference. Given a pre-specified confidence change for a particular experiment and the average length of peptide overlapping the region, we calculate the following table. We use the fact that to measure at 95% confidence the difference must be greater than twice the standard deviation and at 99% confidence the difference must be greater than three times the standard deviation. The inferred value of Sigma measures the standard deviation and we quote results for typical lengths of peptides (5,10,15), with linear dependence on the length of the peptide. Ultimately, there is a complex relationship between redundancy structure, peptide-level variability, redundancy number, peptide length and replicate count that contribute to whether an effect to detected or not. For example, with typical measurement precision (Sigma = 0.001) and moderate peptide length (10 residues), REX can detect changes as small as 1% deuterium uptake difference at 95% confidence.

Minimum Detectable effects (delta HDX)						
Peptide Length	5	10	15	5	10	15
Confidence level	95%	99%	95%	99%	95%	99%
Sigma						
0.001	0.01	0.02	0.03	0.015	0.03	0.045
0.002	0.02	0.04	0.06	0.03	0.06	0.09
0.003	0.03	0.06	0.09	0.045	0.09	0.135
0.005	0.05	0.1	0.15	0.075	0.15	0.225
0.01	0.1	0.2	0.3	0.15	0.3	0.45

5 Structural annotations distributions

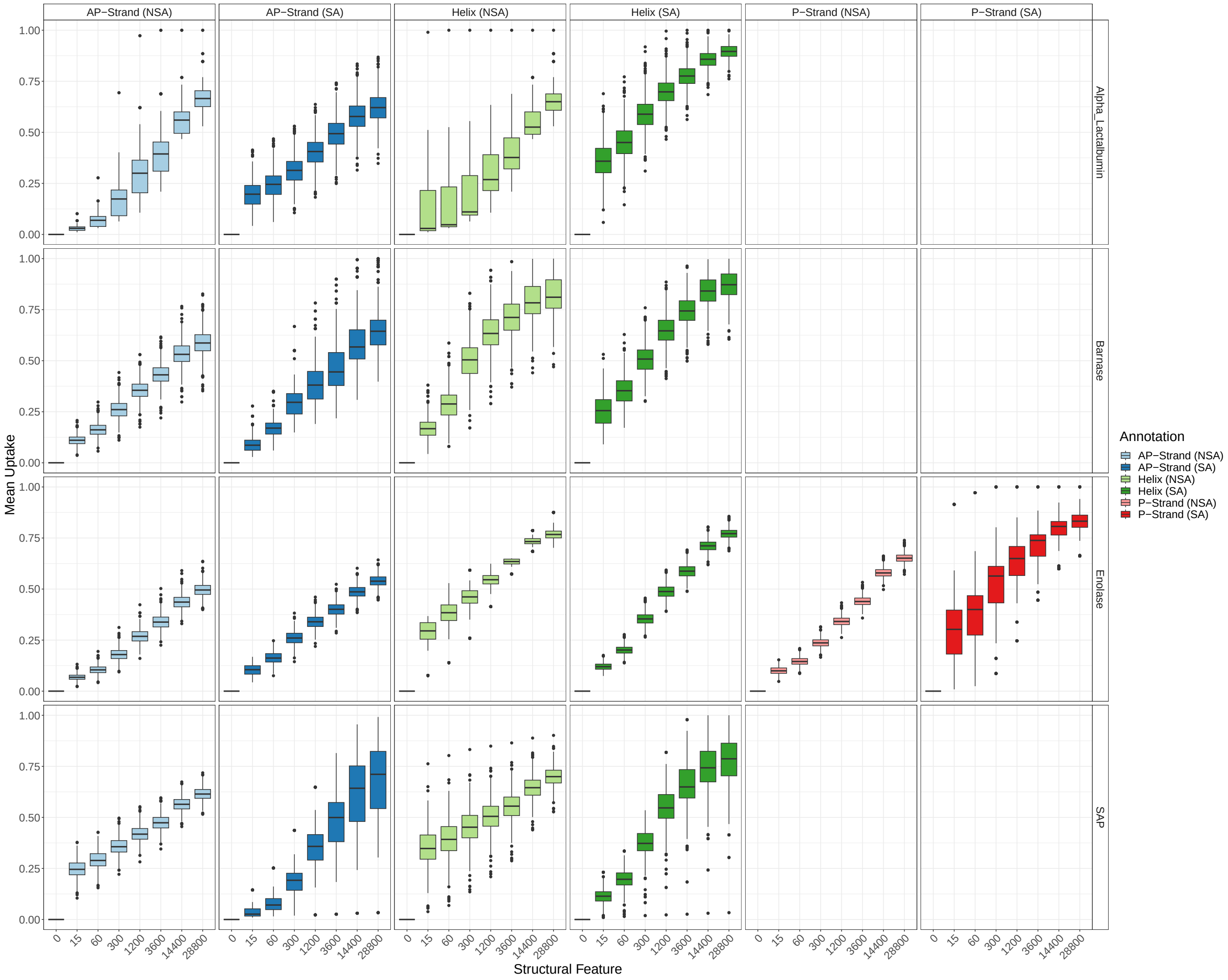

Figure S5: **Structural Features Boxplots.** Box plots corresponding to main Fig. 4B. The distribution are over random subsamples, are x-faceted by structural feature and y-faceted by protein.

6 Differential HDX-MS simulations Meta-Analysis

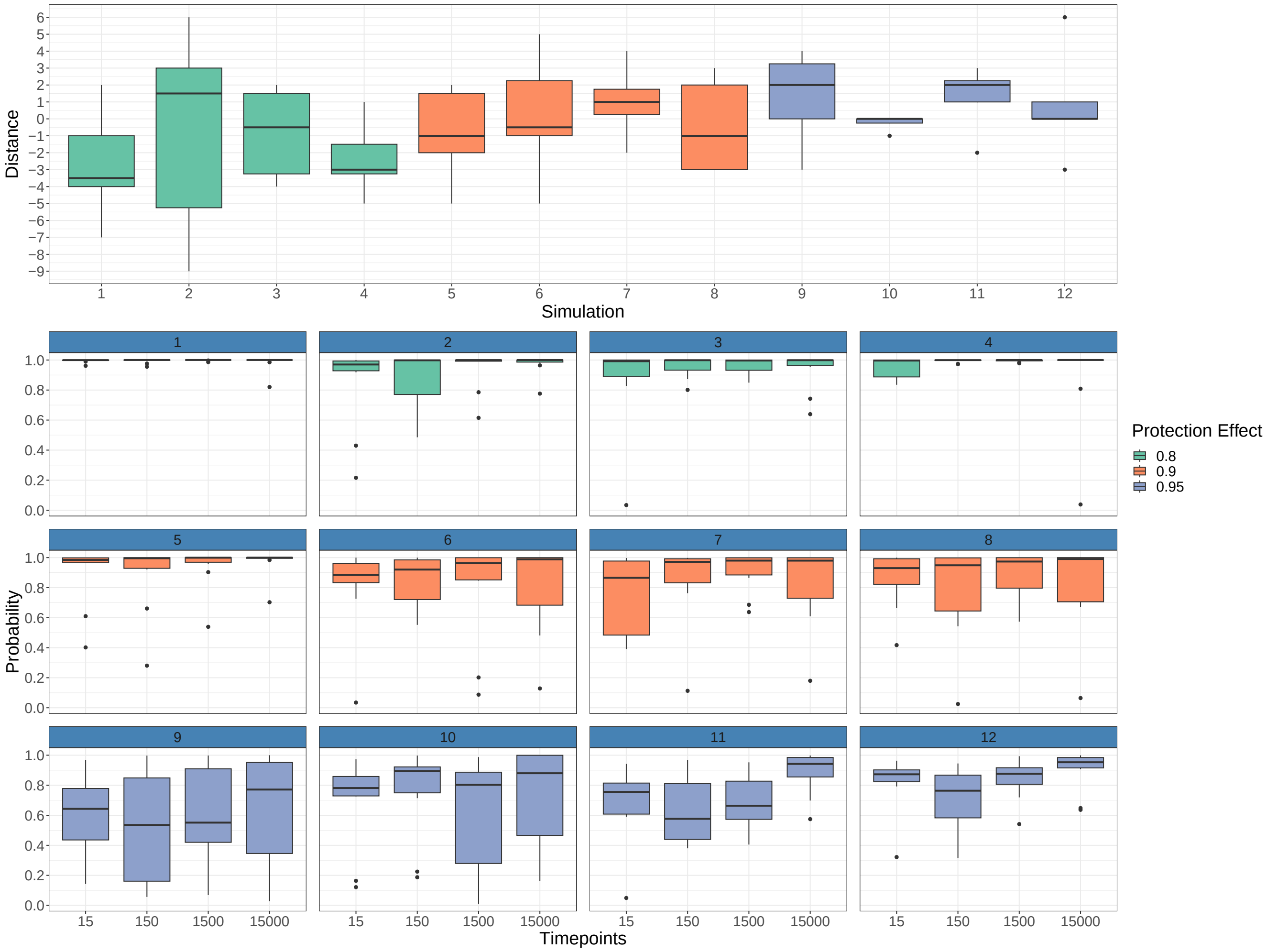

Figure S6: **Meta-analysis of simulations.** Complete results of simulation study. Simulations are index one through twelve with each simulation repeated over ten random seeds. Upper plot show distance from residue of maximal probability to residue that was selected for perturbation. Lower plot is faceted by simulation and indexed by time points. The boxplots show the probability given to residue selected for the simulation. Note that an 80% protection effect is a 20% reduction in deuterium uptake.

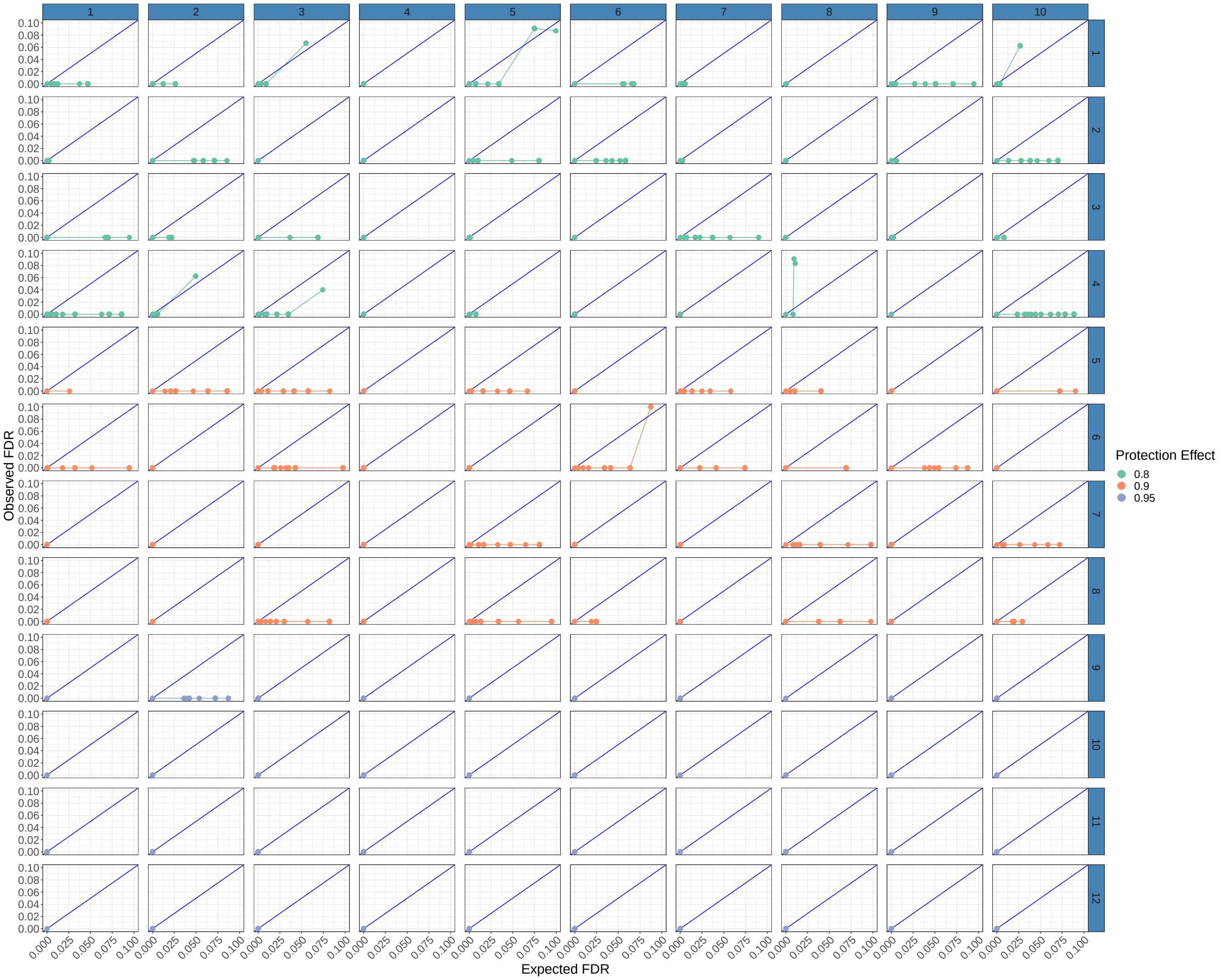

Figure S7: **Calibration plots.** Calibration plots showing expected FDR against observed FDR. Note that the lines are mostly below the y-x axis. The data is x-faceted by random seed and y-faceted by simulation. Note that not all EFDR levels are computable from the simulations.

To determine the relationship between redundancy (number of peptides that overlap a particular residue) and the quality of our results, we examined whether the distance from the perturbed residue to the residue with highest probability was affected by redundancy. Figure S8 (left) shows that this quantity does not vary with redundancy. We also examined whether the probability associated with the perturbed residue varied with redundancy (right). Again we found no relationship between the two quantities. Based on our analysis, while redundancy of 1 can work, we recommend a minimum redundancy of 2-3 for reliable results in most experimental conditions.

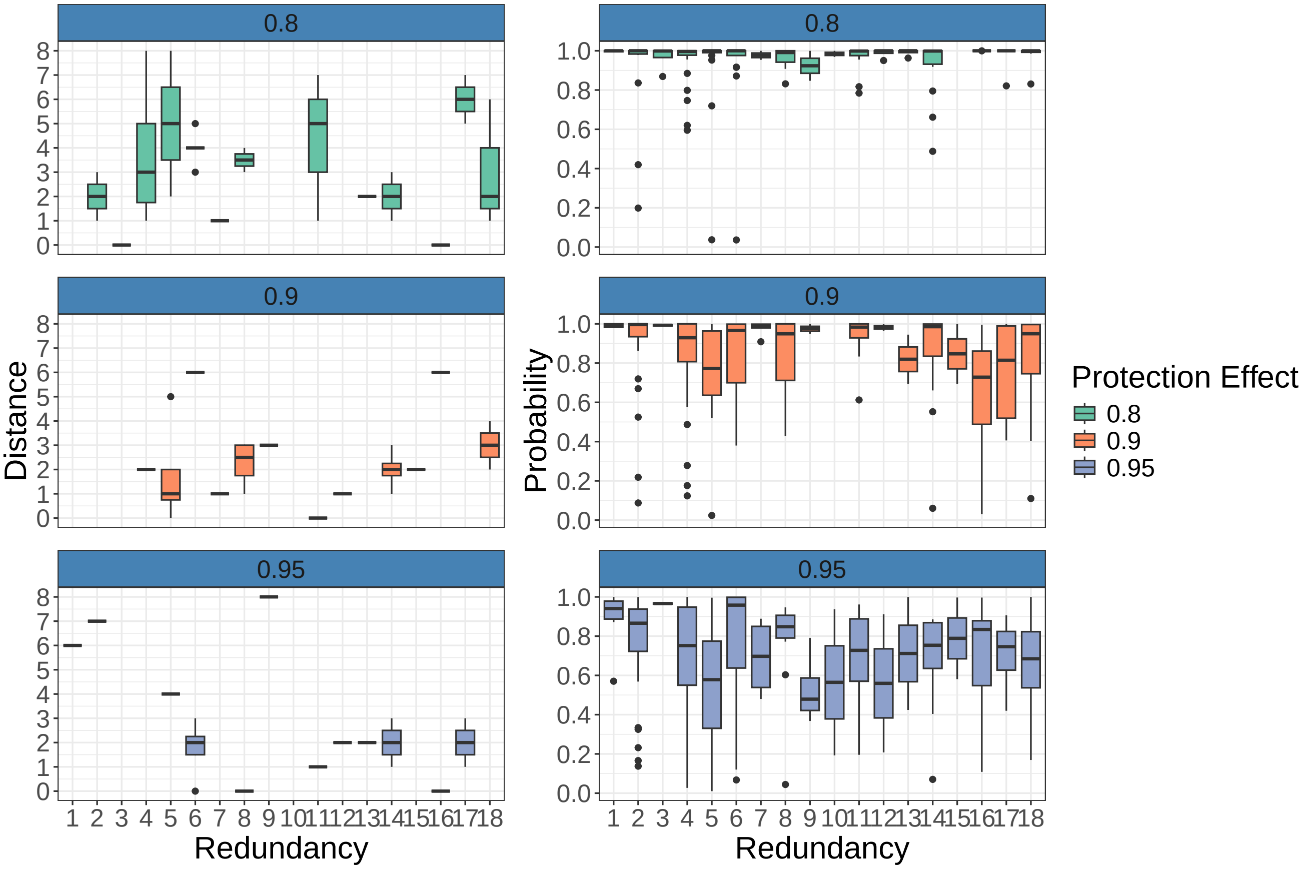

Figure S8: **Redundancy.** Boxplots demonstrating the relationship between redundancy and metrics computed in meta-analysis. No statistical relationship is observed using a linear model.

7 Prior Sensitivity Analysis

We performed a prior sensitivity analysis to understand how prior choices influenced the results. Whilst we could not consider all possible combinations, we selected a handful of prior parameters and considered combinations of these prior options. We chose parameters that we thought would have a large effect on the final outcomes. The parameter choice were $\lambda \in \{1, 10, 100\}$, $m \in \{-5, -4, -3, -2\}$, $p_{\text{shape}_2} \in \{50, 500\}$, $b_\beta \in \{20, 200, 2000\}$. We considered all combinations of these options along with our defaults resulting in 72 different parameter combinations. In each scenario, we summarised the results by computing the number of change points, the value of σ, and the maximum values of ARE and TRE indicated error levels. To increase prior sensitivity we reduced our Cytochrome C to a single replicate and focused on the first 50 residues. The full results table can be found in the Supplementary Data.

We first examined how the number of change points affected prior choices. In Fig. S9 A, we see that increasing λ, the parameter that controls the prior on the Poisson process, increases the number of change points as expected. We also see that increasing b_β results in a decrease to the number of change points. This is because increased values of b_β encourages higher rate of exchange forcing uptake values close to 1 for all timepoints.

We then examined how prior choices were reflected in the inferred value of σ. In Fig. S9 B, we see that increasing λ, has no effect on the values of Sigma. However, there is a clear, as expected, positive relationship between the m, the prior mean on $\log \sigma$, and the values of σ (on the log scale). Given that m is a prior directly on $\log \sigma$ this is the intended effect of the prior.

Ultimately, how these prior combinations affect modelling accuracy is more complex. In Fig.S9 C, we plot the maximum values of the ARE (Max ARE) which measures modelling accuracy. We see that as the number of changepoints increases then Max ARE increases. This suggests that whilst a larger number of changepoints leads to more granular results, it is also potentially allowing the model to overfit. We find that lower errors are generally found for lower number of change points (smaller lambda), middle values of b_β, and lower values of m. Hence, we recommend avoiding prior combinations that lead to extreme uptake values and a large number of changepoints.

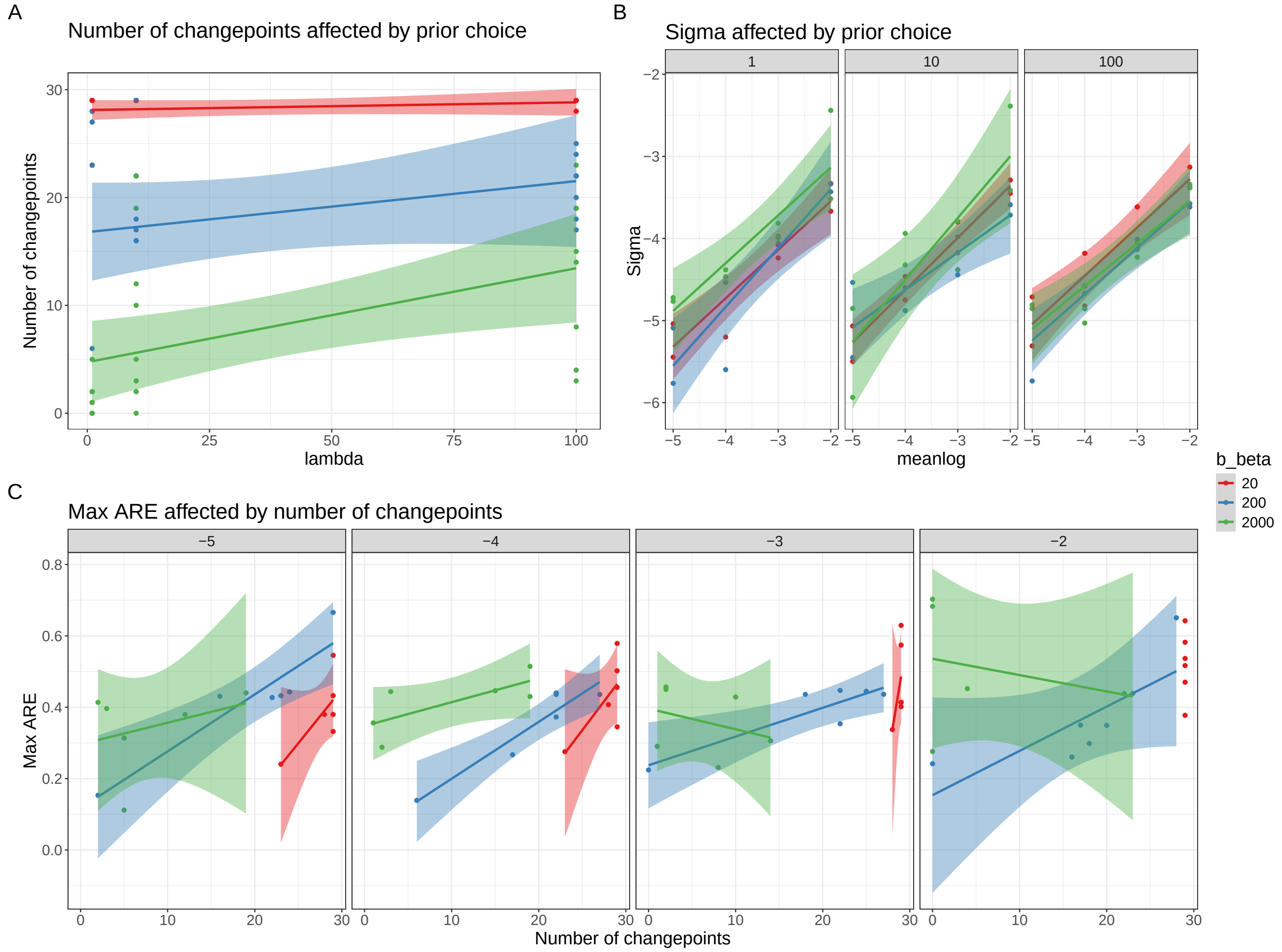

Figure S9: **Prior Sensitivity** (A) The number of changepoints affect by the lambda parameter, coloured by the value of b_β. (B) The value of $\log \sigma$ is affected by the value of m faceted by the values of lambda and coloured by values of b_β (C) Maximum Average Reconstruction Error (Max ARE) plotted against the number of changepoints faceted by the value of m and coloured by the value of b_β.

8 Additional LXRα analysis

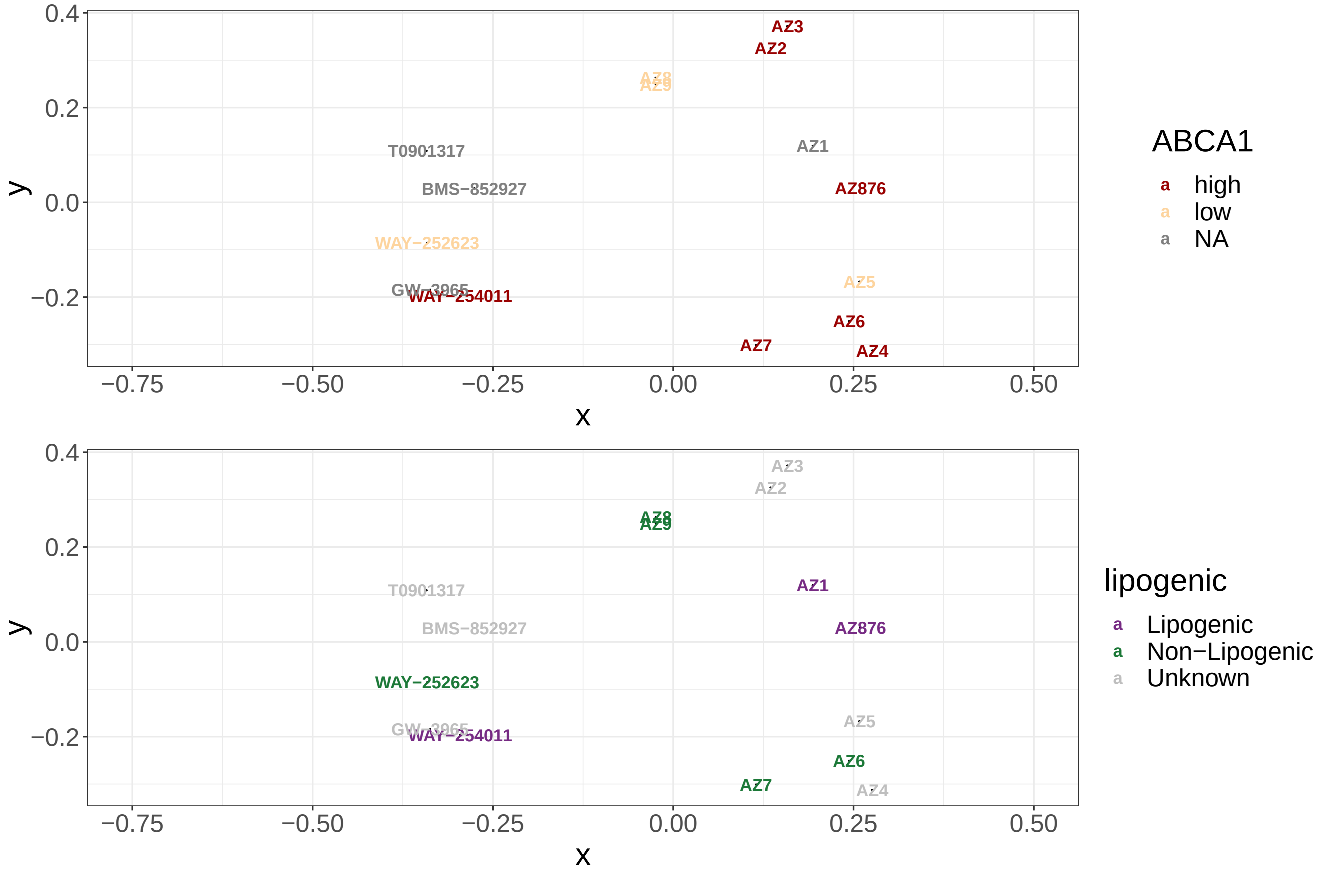

Figure S10: **Multidimensional scaling of chemical similarity** Each figures show a Multidimensional scaling (MDS) scaling plot the separation of the ligands in chemical (Tanimoto) similarity. Neither x nor y components separate in vivo determined functions. Compounds that induce conformationally distinct profiles cluster with similar compounds. Indicating conformation is more closely linked to function that chemical similarity.

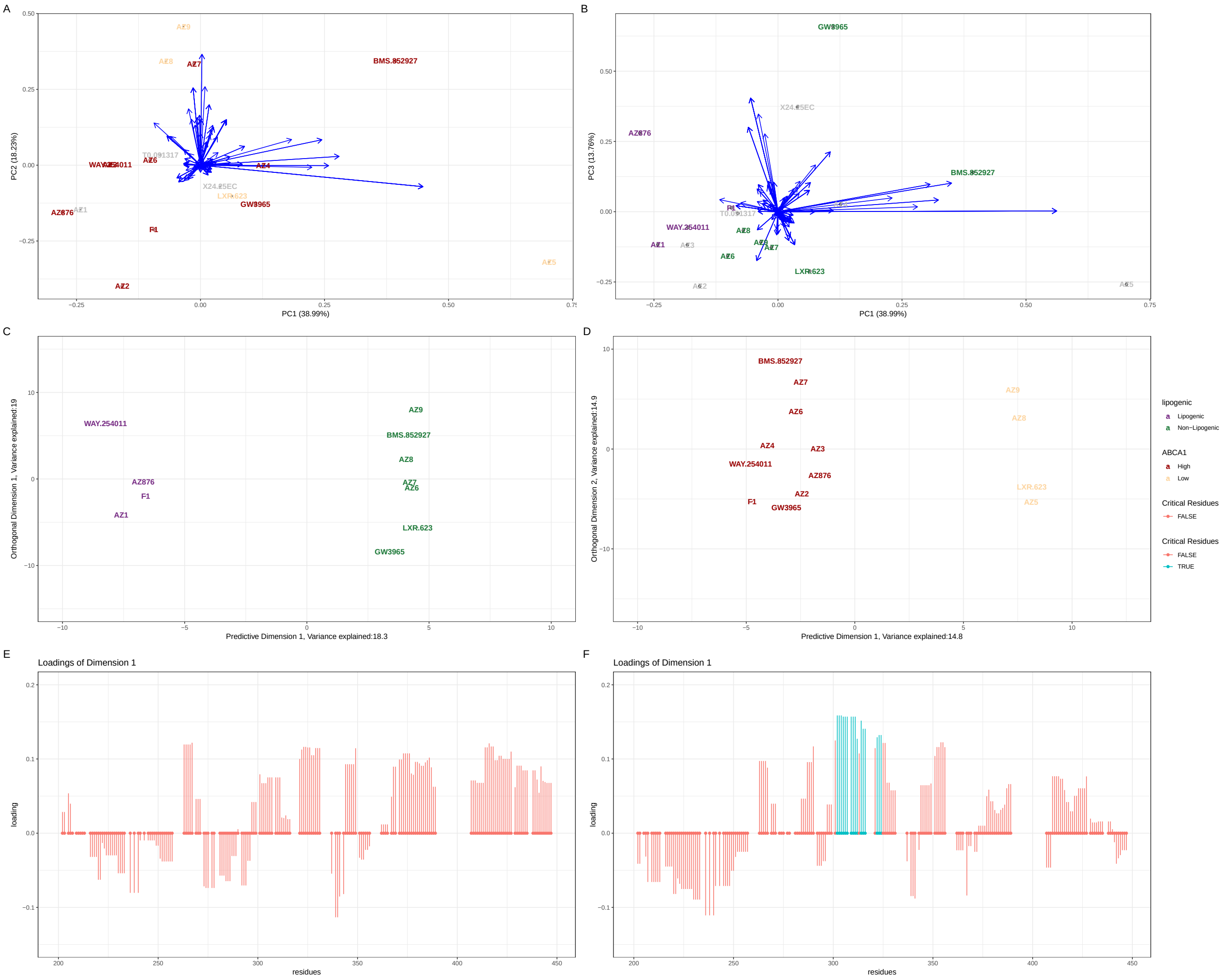

Figure S11: **LXRα in complex with SRC1 CSA.** (A) PCA plots of HDX-MS signatures per ligand. Ligands are coloured by ABCA1 induction and blue arrow represent residue contributions. (B) As with (A) but for PC1 versus PC3. The ligands are coloured by lipogenic annotation. (C) An OPLS-DA plot with lipogenic as the outcome variable (D) An OPLS-DA plot with ABCA1 induction as a outcome variable (E) The loading plot corresponding to the predictive dimension of (C) larger loadings indicate greater contribution. (F) A loading plot corresponding to the predictive dimension of (D).

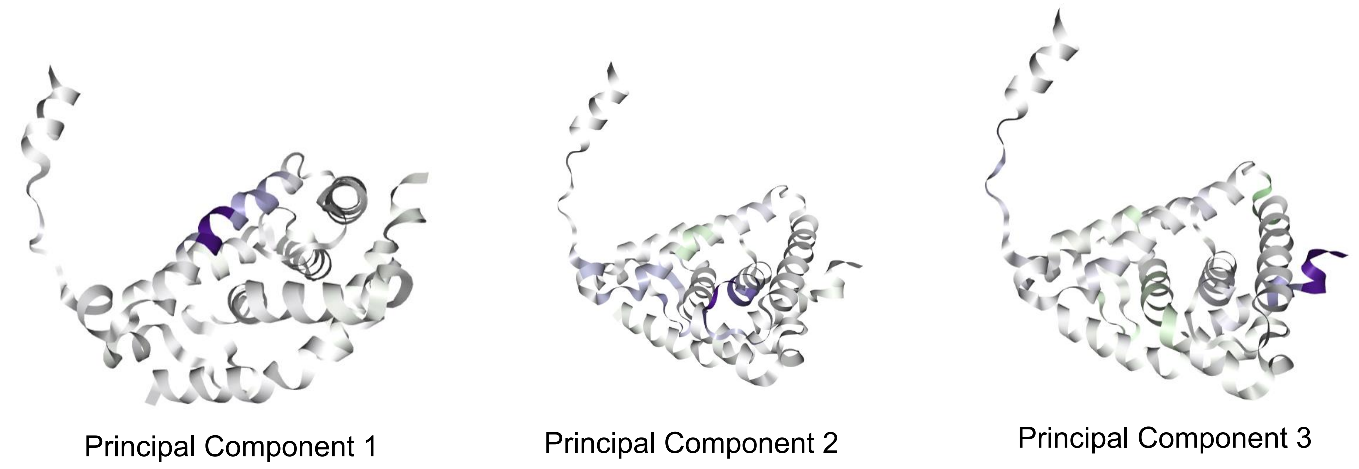

Figure S12: **LXRα in complex with SRC1 CSA PCA loadings.** Loadings corresponding to the annotated principle components from Fig. S11 A and B. Plotted directly on the structure of LXRα

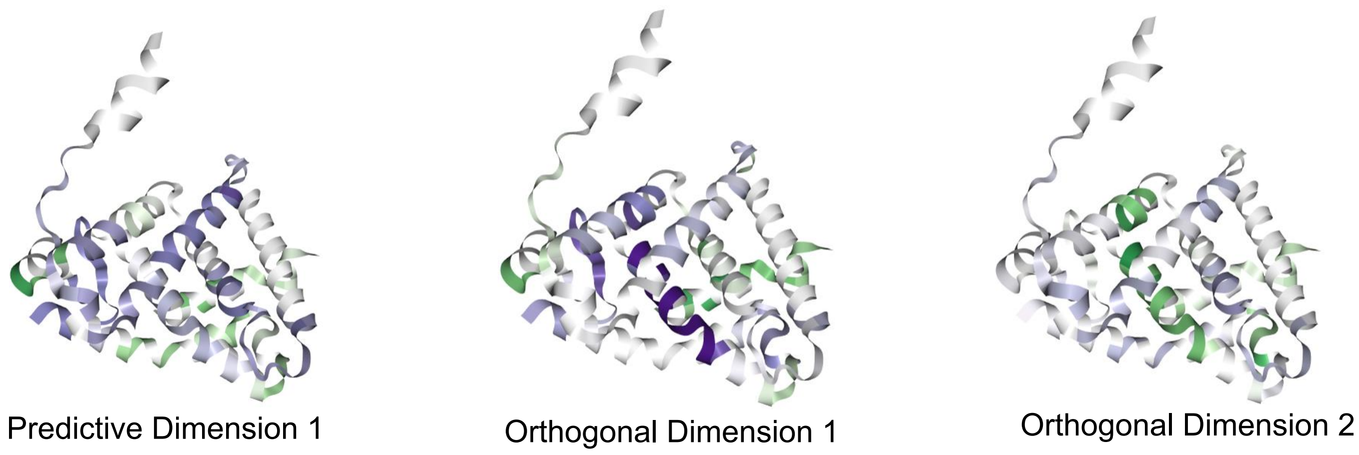

Figure S13: **LXRα in complex with SRC1 CSA OPLS-DA loadings.** Loadings corresponding to the annotated OPLS-DA from Fig. S11 A and B. Plotted directly on the structure of LXRα

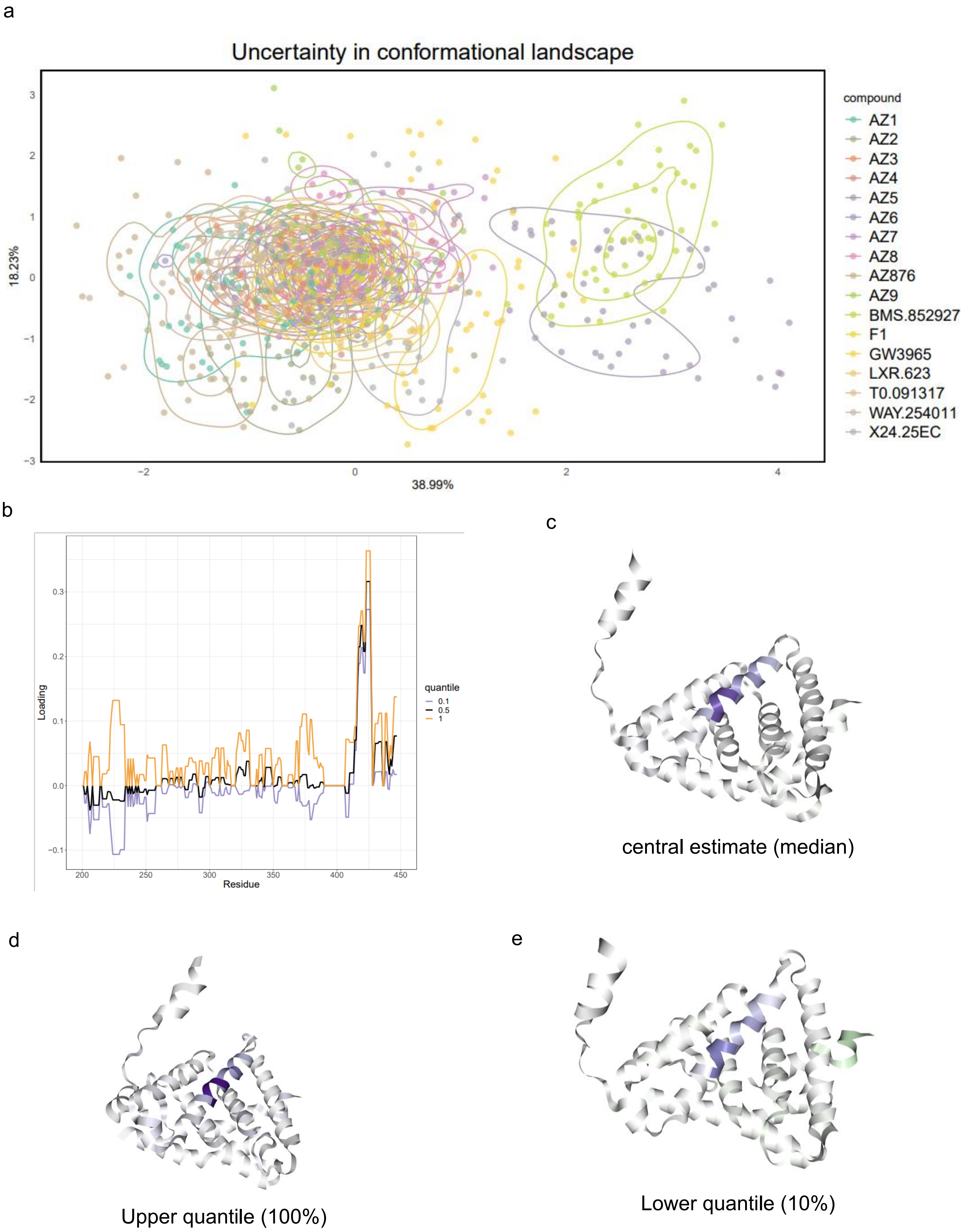

Figure S14: **LXRα in complex with SRC1 CSA Uncertainty Quantification.** (A) A PCA (PC1 vs PC2) plot with uncertainty contours representing the posterior distribution of the locations of molecules in PCA coordinates. (B) The corresponding quantiles in the loadings of the principal components (C) The loading plotted directly on the protein structure corresponding to the mean estimate of the loadings. (D) As with (C) but the upper quantile of the loading (E) as with (D) but with the lower quantile of the loadings.

9 BRD protein alignments

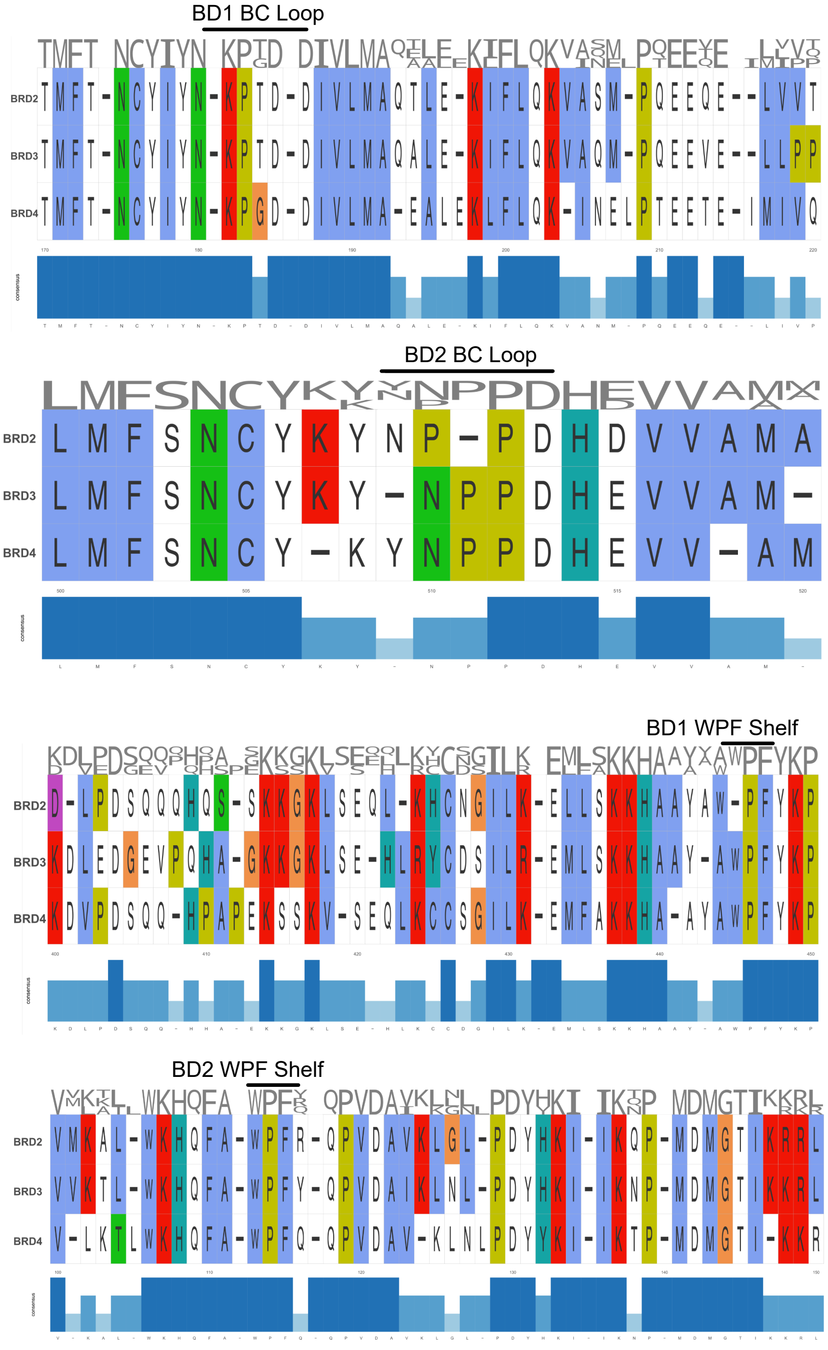

Figure S15: **BRD 2,3 and 4 sequence alignment** Sequence alignments (Clustal) for BRD2,3 and 4 focusing on the BC loop and WPF shelf.

10 Structure Evaluation from HDX

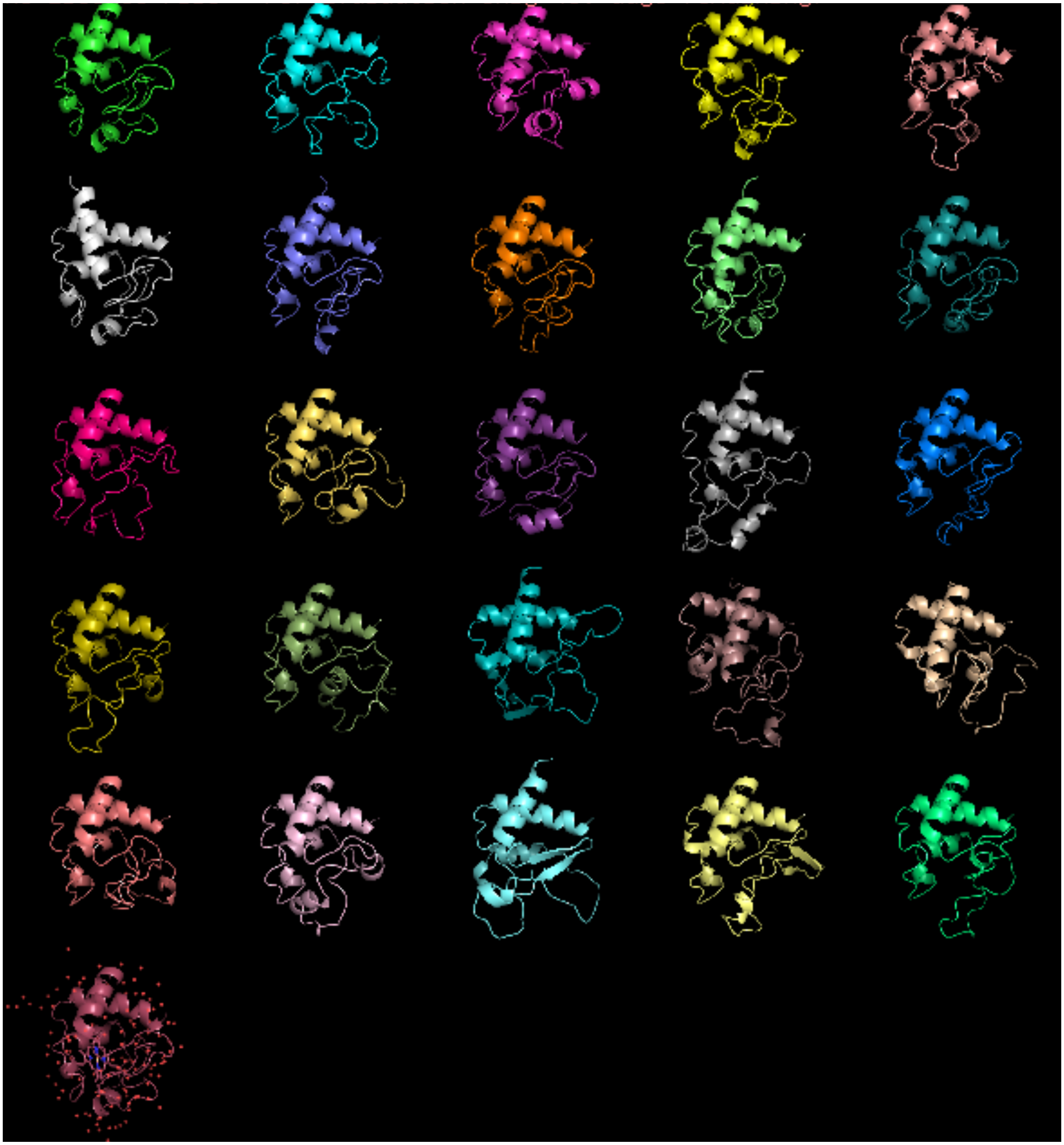

Figure S16: **AlphaFold models of Cytochrome C.** AlphaFold (AF) model of Cytochrome C generated using multiple sequence alignment (MSA) subsampling (Wayment-Steele *et al.*, 2022). The models are wrapped row-wise in order and the crystal structure is given for reference. Note that heme is in the crystal structure (PDB: 1HRC) and this co-factor is not used to generate the AlphaFold structures. Cytochrome C is also in AF trainings set.

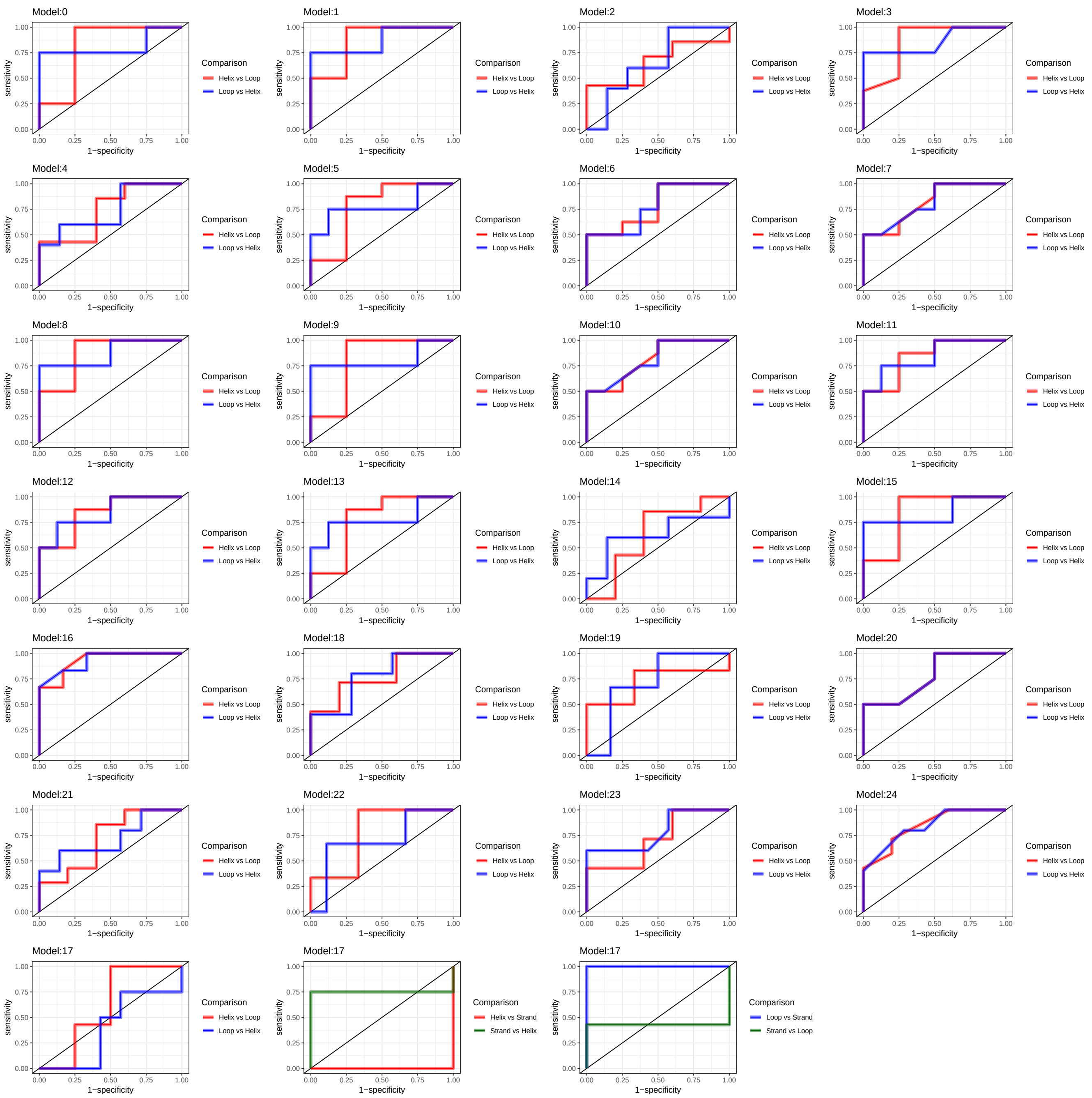

Figure S17: **ROC curves for Secondary Structure prediction.** ROC curves showing predictive performance of HDX feature in predicted secondary structure elements. Curves are shown for both prediction directions. A holdout dataset of 20% of residues is used for each predicted model.

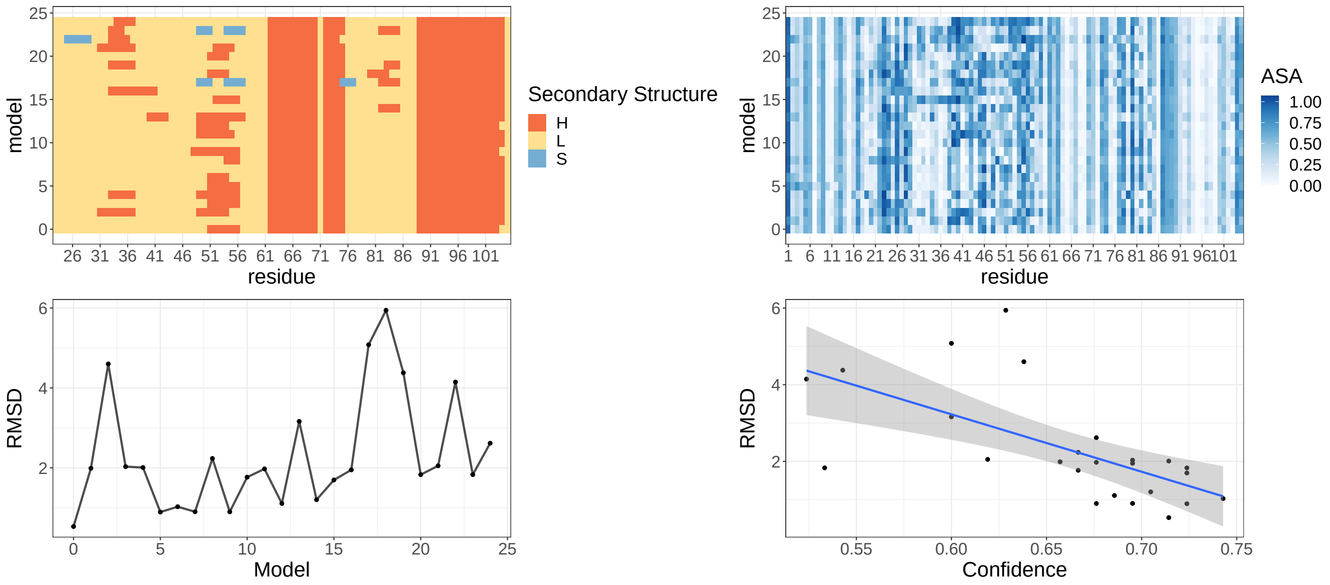

Figure S18: **Evaluating confidence in AlphaFold models.**(Top left) Secondary Structure annotations for each model. (Top right) Accessible solvent area for each model. (Bottom left) RMSD to crystal structure of each model. (Bottom right) RMSD as a function of random forest derived confidence. Higher confidence leads to lower RMSD. Linear model shown with standard error confidence band.

11 Supplementary methods

11.1 Simulation Study

Here, we provide additional clarification the simulation experiments used in the main text. The simulation study consider the four following steps:

1. Randomly select a residue from the protein.

2. Identify all peptide overlapping that residue.

3. Reduce deuterium percentage of those overlapping residues by specified percentage .

4. Apply Rex methodology.

The simulation experiment is visualise in figure S19. Despite only simulating protection effects

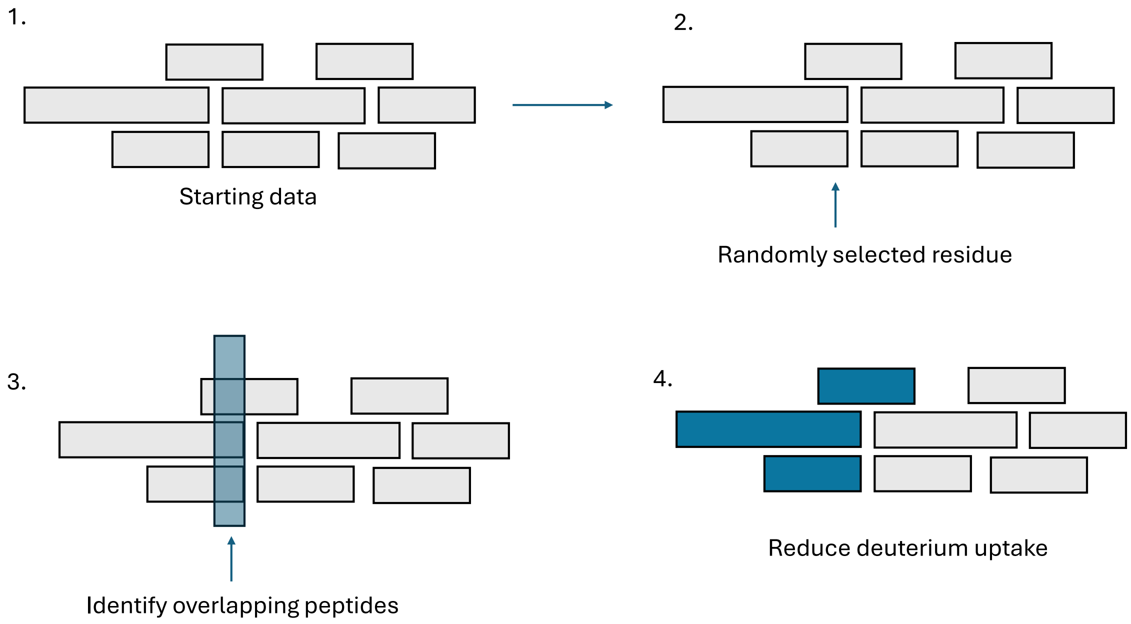

Figure S19: **Visual explanation of simulation study** The figure clarifies the simulation experiments in the main text.

by reducing deuterium uptake, we (perhaps surprisingly) expect to see de-protection effects and protection effects in other parts of the protein with this simulation set-up. This is a subtle and overlooked problem. If a peptide displays a protection effect (reduction in deuterium uptake) but overlapping residues display no change (zero reduction in deuterium), then since the uptake for a peptide is the sum of individual residues, if we are to maintain the zero sum for that overlapping peptide there must be a positive (increase in deuterium uptake) effect to compensate. This is despite no de-protection effect at the level of peptides, hence we believe there are many "silent"

effects at the peptide-level. If neighbouring peptides also have no change this silent effect simply propagates unseen at the residue level. This is because the unseen de-protection effect must be then balanced by a corresponding protection effect. This effect is observed in our analysis and figure S20 demonstrate this phenomenon visually.

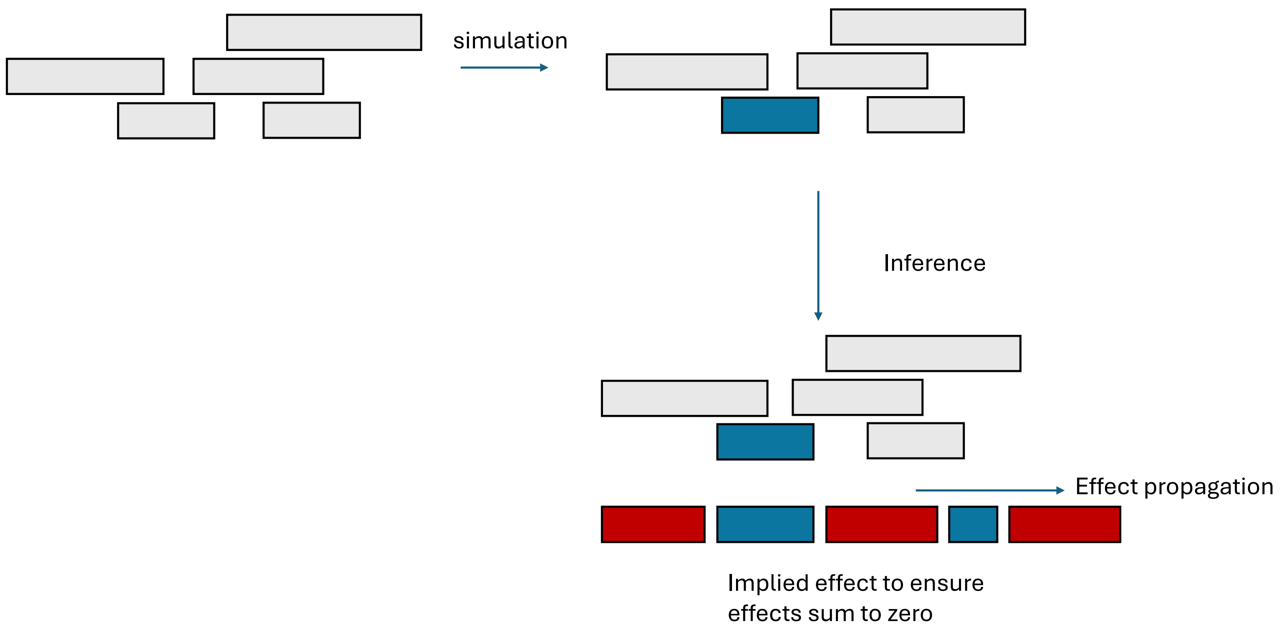

Figure S20: **Visual explanation of protection propagation effect** The figure explain the protection propagation effect.

11.2 AF-Cluster

AlphaFold conformation were generated as described in Wayment-Steele *et al.* (2022) with version dated Jan 2023 from the Google collab notebook. In particular a multiple sequence alignment (MSA) of Cytochrome C was generated and clustered. The method was run with default settings with three recycles and model number set to three. This generated a total of 25 candidate models.

11.3 Secondary structure assignment

The Secondary structure of Cytochrome-C was re-annotated using STRIDE (Heinig and Frishman, 2004).

11.4 Prediction of secondary structure

The Secondary structure was predicted per model using a random forest model with an 80/20 split between training and testing.

11.5 Accessible surface area

Relative ASA was calculated using FreeSASA (Mitternacht, 2016) using the standard spherical probe model.

11.6 Confidence scores

Root mean squared deviation (RMSD) calculations were made between each model to the crystal structure (PDB: 1HRC). Using a random forest model we predicted both secondary structure and accessibility (ASA > 0.25) with a 80/20 residue train/test split. The prediction probabilities for secondary structure and ASA were obtained from the model and a per residue confidence score was calculated by multiplying these probabilities together. A per model confidence score was obtained by computing the proportion of residue confidence scores above 0.5.

11.7 MCMC convergence

We use the Gelman-Rubin diagnostic (Gelman *et al.*, 2013) on 4 parallel chains to assess convergence of the RJCMCMC algorithm. 5,000 steps were performed each with 5 Reversible-Jump moves per step (e.g. 25,000 Reversible-Jump moves in total). Chains were inspected manually for convergence, removing uncovered chains and removing the first 3,000 iterations for burn-in. We then confirmed that the Gelman-Rubin diagnostic was close to 1 on the log likelihood and Sigma parameters. We found BRD4 converged faster, running only 2,500 steps (12,500 Reversible-Jump Moves), using 1,500 steps for burn-in. Illustrative plots are shown below:

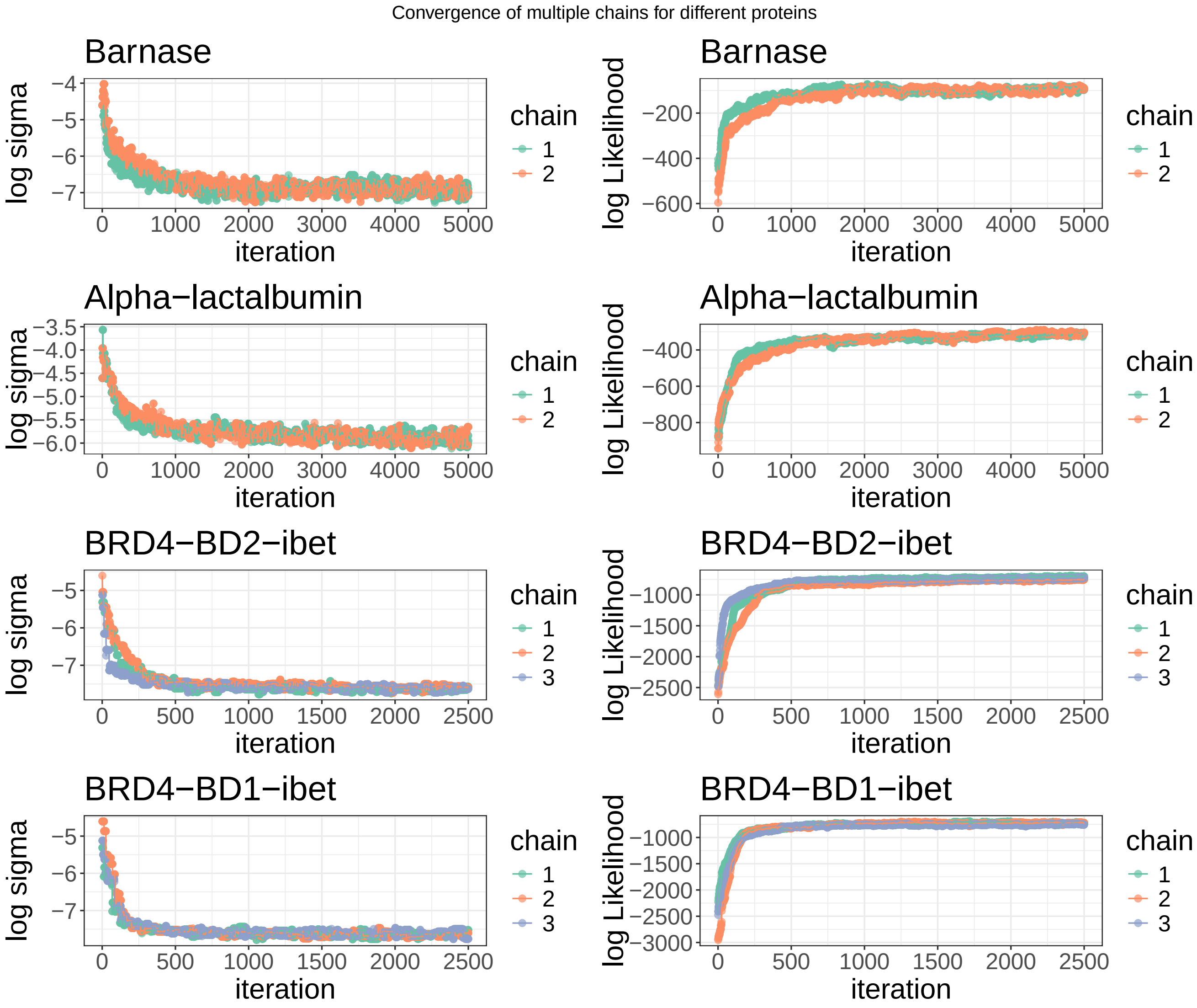

Figure S21: **MCMC trace plots to check convergence** The figure shows example MCMC trace plots.

References

Gelman, A. et al. (2013). *Bayesian data analysis*. CRC press.

Heinig, M. et al. (2004). Stride: a web server for secondary structure assignment from known atomic coordinates of proteins. *Nucleic acids research*, **32**(suppl_2), W500–W502.

Mitternacht, S. (2016). Freesasa: An open source c library for solvent accessible surface area calculations. *F1000Research*, **5**.

Moulick, R. et al. (2015). Partially unfolded forms of the prion protein populated under misfolding-promoting conditions: characterization by hydrogen exchange mass spectrometry and nmr. *Journal of Biological Chemistry*, **290**(42), 25227–25240.

Stofella, M. et al. (2022). High-resolution hydrogen–deuterium protection factors from sparse mass spectrometry data validated by nuclear magnetic resonance measurements. *Journal of the American Society for Mass Spectrometry*, **33**(5), 813–822.

Wayment-Steele, H. K. et al. (2022). Prediction of multiple conformational states by combining sequence clustering with alphafold2. *BioRxiv*, pages 2022–10.
