# Supplementary material for: Inferring residue level hydrogen deuterium exchange with ReX
Source: Commun Chem. 2025 Nov 10;8:343. doi: 10.1038/s42004-025-01719-4 (PMC12603136; doi:10.1038/s42004-025-01719-4)
Supplement: Supplementary file 3 — Description of Additional Supplementary Files [file 42004_2025_1719_MOESM3_ESM.pdf]

Description of Additional Supplementary Files

File name: Supplementary Data 1

Description: Cytochrome C HDX-MS data with Nepenthesin II digestion

File name: Supplementary Data 2

Description: Cytochrome C HDX-MS data with Pepsin digestion

File name: Supplementary Data 3

Description: Cytochrome C HDX-MS data with Type 13 digestion
